# *Ex vivo* near-infrared targeted imaging of human bladder carcinoma by ICG-anti-CD47

**DOI:** 10.3389/fonc.2023.1083553

**Published:** 2023-03-01

**Authors:** Haifeng Hao, Xinyu Wang, Yan Qin, Zhifang Ma, Pengyu Yan, Chao Liu, Guanying Chen, Xiaofeng Yang

**Affiliations:** ^1^ Department of Urology, First Hospital of Shanxi Medical University, Taiyuan, China; ^2^ School of Chemistry and Chemical Engineering, Harbin Institute of Technology, Harbin, China; ^3^ Department of Gastroenterology, Shanxi Provincial People’s Hospital, Taiyuan, China

**Keywords:** bladder carcinoma, indocyanine green, CD47, near-infrared fluorescence, molecular imaging

## Abstract

**Objective:**

The low detection rate of early-stage and small tumors remains a clinical challenge. A solution to this unmet need is urgently warranted for the accurate diagnosis and treatment of bladder cancer (BC). This study aimed to evaluate the feasibility of CD47 as a target for optical molecular imaging of human BC and conduct preliminary *ex vivo* imaging experiments.

**Method:**

Using indocyanine green (ICG) and a CD47 antibody (anti-CD47), we synthesized a new targeted fluorescent probe ICG-anti-CD47. A total of 25 patients undergoing radical cystectomy were prospectively included in *ex vivo* imaging experiments. Following surgery, the freshly isolated bladder specimens were incubated with ICG-anti-CD47, and images were captured under white light and near-infrared (NIR) light. Standard histopathologic evaluation was performed, and findings were correlated with those of CD47-targeted NIR molecular imaging.

**Results:**

Based on the *ex vivo* imaging experiments, 23 and 2 patients were pathologically diagnosed with bladder urothelial carcinoma and bladder squamous cell carcinoma, respectively. There were no adverse effects of ICG-anti-CD47 on the histological structure of the tumor and normal uroepithelium. In the NIR grayscale images, the mean fluorescence intensity of the tumor tissue was significantly higher than that of the adjacent normal background tissue, which markedly improved tumor visualization.

**Conclusion:**

Anti-CD47-targeted NIR molecular imaging may be a feasible and powerful strategy for the accurate diagnosis of BC. Nevertheless, larger-scale randomized trials are warranted to verify the present findings.

## 1 Introduction

Bladder cancer (BC) is the tenth most frequent type of cancer worldwide and the second most common malignant disease of the urinary system (1). It accounts for approximately 474,000 new cases and 197,000 deaths per year. Non-muscle invasive BC (NMIBC) (stages: Ta, carcinoma *in situ* [CIS], and T1) accounts for approximately 75% of newly diagnosed BC cases. International guidelines recommend that transurethral resection of bladder tumor (TURBT), coupled with individualized intravesical chemotherapy or immunotherapy that is adapted to tumor risk stratification, should be administered to those patients as the routine treatment regimen (2–4).

BC has the highest recurrence rate among all types of cancer, reaching 61% within 1 year (5). Approximately 20% of patients with high-risk NMIBC develop muscle-invasive bladder cancer (MIBC) within 5 years (6). Neoadjuvant chemotherapy combined with radical cystectomy is the first-line treatment for MIBC, and the tumor-specific survival rate of patients after 4 years is 35% (7). Therefore, it is important to optimize the detection and resection of early-stage BC.

The recurrence and progression of bladder tumors endanger patients’ lives, and repeated surgical intervention increases medical costs and negatively influences the well-being of patients. In recent years, these effects of BC have attracted considerable attention (8, 9). Thus, appropriate management of BC is crucial to prolong the overall survival of patients and reduce medical costs. Currently, light cystoscopy (WLC) and WLC-aided TURBT are the standard methods used for the detection, resection, and monitoring of BC. Nevertheless, these techniques are limited by the risk of missed diagnosis, insufficient resection, and inaccurate staging (10).

The bladder can be easily accessed, providing a well-established route for local (i.e., intravesical) drug delivery. Intravesical administration can minimize the potential systemic toxicity of imaging agents, rendering the bladder a suitable organ for endoscopic molecular imaging and image-guided surgery. Recently, attempts have been made to improve the specificity and sensitivity of methods used for the detection of malignant lesions. Consequently, the traditional imaging mode based on functional and structural changes has progressively transformed into molecular imaging based on cellular and molecular pathological processes (11). An increasing number of auxiliary optical imaging technologies have been applied to enhance WLC (12). Optical molecular imaging is the integration between new optical imaging technology and modern molecular biology. The attachment of targeted fluorescent tracers with overexpressed molecules on tumor cells or tumor microenvironment enables qualitative and quantitative analyses of tissue biological behavior in terms of molecular and cellular alterations before the occurrence of changes in the macroscopic structure of tissues (13). Paired optical imaging devices can reveal small or occult tumors with a low rate of false-positive results. Unlike ultrasound or magnetic resonance imaging, optical molecular imaging can provide real-time dynamic images during surgery and be applied to numerous aspects of cancer surgery. These include the detection of small, flat malignant lesions, access to the boundary and depth of tumor invasion, and visualization of important anatomical structures (14). A CD47 antibody (anti-CD47) has been utilized in targeted imaging and tumor-specific administration for bladder urothelial carcinoma (15). CD47-targeted optical molecular imaging can detect BC with high diagnostic accuracy and has the potential to guide its resection (16).

Using indocyanine green (ICG) and anti-CD47, we synthesized a new ICG-anti-CD47 near-infrared (NIR) fluorescence probe for the diagnosis of bladder carcinoma in fresh human samples obtained through radical cystectomy. This report identified a series of diagnosis and treatment alternatives. We performed *in vitro* NIR molecular imaging of 25 newly isolated BCs by intracavitary perfusion of NIR fluorescent dye ICG-anti-CD47 and ICG. The results of this study may provide a foundation for the clinical use of CD47 as a molecular imaging target for BC diagnosis and treatment, particularly bladder CIS.

## 2 Materials and methods

### 2.1 Synthesis of amino-modified ICG dots

ICG and amino-modified 1,2-distearoyl-sn-glycero-3-phosphoethanolamine-N- methoxy(polyethylene glycol)-2000 (DSPE-PEG-NH2) were dissolved in chloroform as stock solutions at a concentration of 1 mg/mL. Subsequently, stock solutions of ICG and DSPE-PEG-NH2 were mixed at a 1:6 volume ratio and sonicated for 1 min. Following further dilution with an additional 10 mL of chloroform, the mixture was dried under a vacuum in a rotary evaporator at 37°C to form a lipidic film. After completely removing the organic solvents, phosphate-buffered saline (PBS) (5 mL; pH 7.4) was added, and the lipidic film was sonicated for 30 min to obtain the clear aqueous solution of ICG-NH2 dots.

### 2.2 Synthesis of ICG-NH2-anti-CD47 dots

Anti-CD47 was loaded on the surface of ICG-NH2 dots through an amide reaction. Briefly, anti-CD47 (1 mL, 200 µg/mL, Santa Cruz Biotechnology, USA) was dispersed in 10 mL of ethanol (EtOH) containing 1-ethyl-3-(3-dimethylaminopropyl) carbodiimide hydrochloride (0.03 mmol) and N-hydroxy-succinimide (0.75 mmol). After stirring for 30 min, an aqueous solution of ICG-NH2 dots (2 mL; 10 mg/mL) was added to the EtOH solutions and stirred overnight. The products were collected by centrifugation, followed by several washes to remove excess reactant. The prepared ICG-NH2-anti-CD47 dots were dried and re-dissolved in PBS to form clear solutions, which were directly used in experiments.

### 2.3 Absorption and fluorescence measurements

Absorption spectra were recorded using an Agilent Cary5000 UV-Vis-NIR spectrophotometer (Agilent Technologies Inc., Santa Clara, California, USA) in toluene in a quartz cuvette with a path length of 1 cm. The luminescence spectra were obtained using an Edinburgh FLS1000 spectrofluorometer equipped with a Xenon lamp (Edinburgh Instruments Ltd., London, UK).

### 2.4 *In vitro* imaging of bladder specimens

From September 2021 to July 2022, with the approval of the ethics committee of the host institution, patients with bladder urothelial carcinoma who underwent radical cystectomy were prospectively enrolled in this study. The presence of bladder carcinoma in these patients was confirmed by computed tomography or magnetic resonance imaging and cystoscopy prior to the operation. Patients with suspected distant metastasis or lymph node metastasis detected through preoperative examination were excluded from the study. All patients provided written informed consent for their participation in the study, and an experienced urologist performed all operations. Fresh and complete bladder samples were collected immediately after resection. The bladder was rinsed thrice with sterile normal saline. Subsequently, ICG-anti-CD47 (excitation, 780 nm; emission, 810 nm; 20 μg/mL); was diluted with PBS at a ratio of 1:100 to prepare 50 mL of the imaging probe, which was slowly injected into the bladder specimen cavity through an 18 F catheter, or ICG (PBS, pH 7.4). Following incubation at room temperature for 30 min, the samples were rinsed thrice with sterile normal saline to remove the unbound antibody. The washed anterior bladder wall was cut longitudinally and placed under the visible and NIR fluorescence separation and combination imager (SES Co., Taiyuan, China). The imager collected both the visible light information and NIR fluorescence information from the bladder specimens in real time. White light, fluorescent, and fused images were simultaneously displayed on the screen. The positive ICG-anti-CD47 fluorescence was designated as the bright area in the NIR grayscale image regardless of the shooting angle. The mean fluorescence intensity (MFI) of the corresponding tissue was expressed according to the mean gray value of the tumor and the adjacent normal background in the NIR grayscale image. The tumor-to-background ratio was calculated as the ratio of the MFI of tumor tissue to that of normal background tissue. After imaging, the fluorescent area was stained with ink and subjected to the standard histopathological examination by the same pathologist. Histological grading and tumor staging were performed according to the World Health Organization grading system established in 2004 and the TNM grading system established in 2017.

### 2.5 Statistical analysis

Statistical parameters were calculated according to the following equations: RP=TP/(TP+FN); SPC=TN/(TN+FP);where TP is the true positive; TN is the true negative; FP is the false positive; FN is the false negative; TRP is the true positive rate or sensitivity; and SPC is the true negative rate or specificity. Quantitative data corresponding to normal distribution were presented as the mean ± standard deviation, and the t-test was used to evaluate the divergence. Non-normally distributed data were presented as the median (range) and compared using the Wilcoxon rank-sum test. The data were analyzed using R 4.0.3 and GraphPad Prism 8.0 for Windows software. P-values of < 0.05 denoted statistically significant differences. All statistical analyses were two-tailed.

## 3 Results

**Figure 1:**
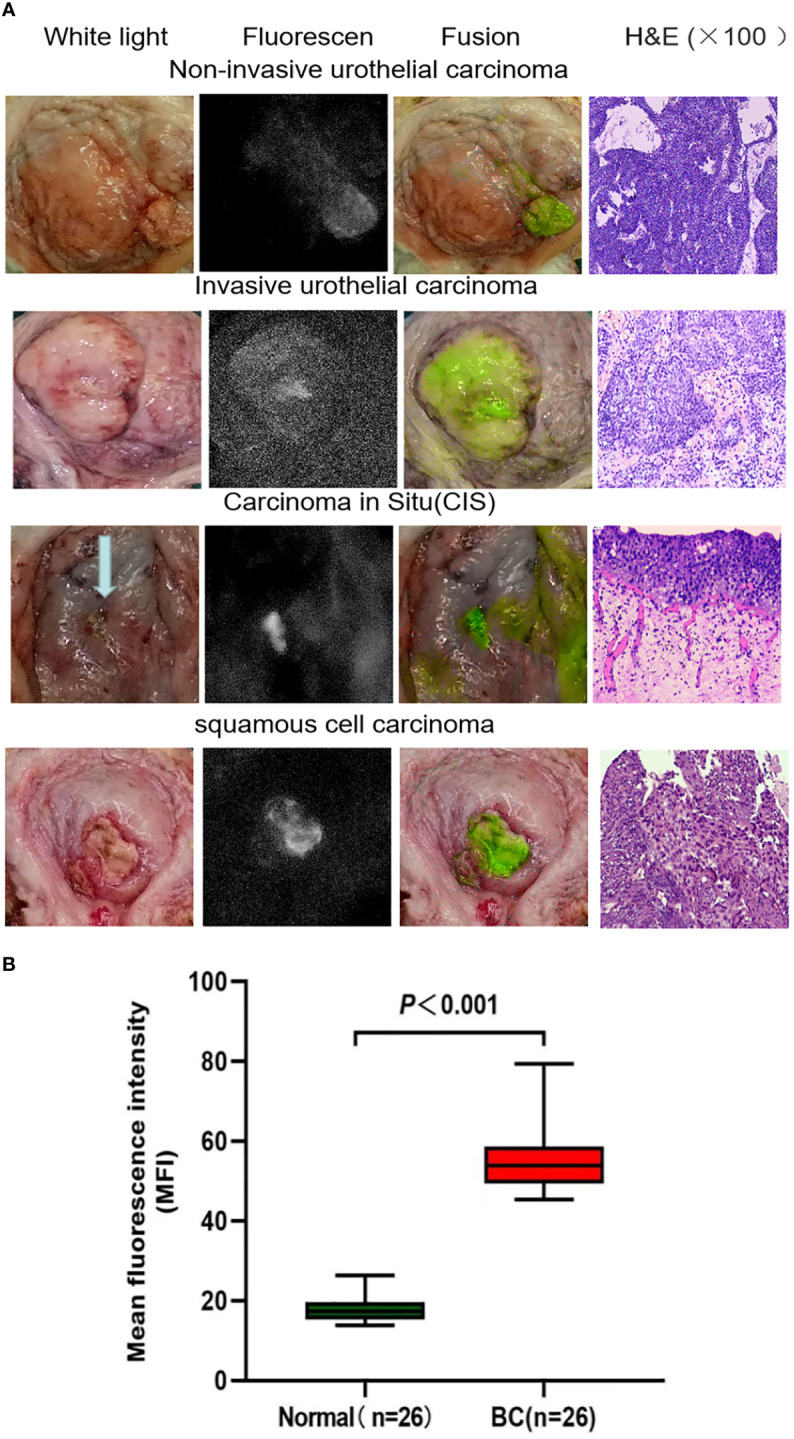
**(A)** Representative images of white light cystoscopy, NIR fluorescence, fusion *ex vivo* imaging of bladder specimens and tumor sections stained with H&E. The images demonstrate the targeting of noninvasive bladder urothelial carcinoma, invasive bladder urothelial carcinoma, carcinoma *in situ*, and bladder squamous cell carcinoma by the ICG-anti-CD47 imaging agent. The diagnosis was confirmed by pathological analysis. **(B)** Quantitative mean fluorescence intensity (MFI) of 22 specimens. The MFI values of tumor tissue and normal tissue were 54.67 ± 16.17 and 19.11 ± 6.41 (P < 0.001), respectively. H&E, hematoxylin-eosin; ICG, indocyanine green; NIR, near-infrared.

In this study, we used anti-CD47 to mark ICG and created a new targeted tracer (ICG-anti-CD47). The fluorescence spectrum of ICG-anti-CD47 had an excitation wavelength of 780 nm. From September 2021 to July 2022, 25 patients (22 males and 3 females) who underwent radical cystectomy were included in the study. The mean age of patients was 66.2 years (range: 43–77 years). **Table 1** shows the patient demographic characteristics, preoperative diagnosis, clinical stages of the disease, and the results of imaging studies. After the incubation with ICG-anti-CD47, there was no detection of any adverse morphological manifestations in the specimens. Additionally, there was no evidence of injury or degenerative effects in non-tumor tissues. The use of ICG-anti-CD47 did not change the pathological evaluation of radical cystectomy. The pathological evaluation of 25 bladder specimens resulted in the detection of 29 malignant lesions, including 22 bladder specimens (26 malignant lesions) stained with ICG-anti-CD47 in the experimental group and 3 bladder specimens (3 malignant lesions) stained with ICG in the control group. Different BC pathological frequencies of 29 cases were as follows: 19 cases of high-grade muscle-invasive urothelial carcinoma, four cases of high-grade non-muscle invasive urothelial carcinoma, four cases of CIS, and two cases of squamous cell carcinoma (**Figure 1A**). In two patients, NIR fluorescence imaging guided pathologists to perform white light examinations of CIS that were not observed. In negative control cases (cases 7, 10, and 17), only ICG was used for perfusion (20 μg/mL concentration; 50 mL volume), and there was no specific tumor targeting. Under NIR light, the fluorescence signal of tumor lesions exhibited a 3.14-fold increase, while the fluorescence signal of surrounding normal tissues did not significantly change (the MFI was 55.47 and 17.94, respectively) (**Figure 1B**). The lesions with significantly elevated MFI were identified as noninvasive papillary urothelial carcinoma according to the histopathological analysis. The areas without ICG-anti-CD47 fluorescence were identified as normal urothelial carcinoma. Sensitivity/specificity results for ICG-anti-CD47 targeting of cancerous lesions in the human bladder specimens: carcinoma vs. normal. The true positive(TP) number is 26, the true negative(TN) number is 24, the false positive (FP) number is 0; the false negative (FN) number is 2. The true positive rate (TRP) or sensitivity is 0.929, and the true negative rate or specificity (SPC) is 1.000.

**Table 1:** Patient demographic information, pathological stage and diagnosis, lesions observed by white light and fluorescence examinations, and results of ICG-anti-CD47 NIR molecular imaging.

Case no.	Sex/age (y)	Pathological stage	Pathological diagnosis	Grade	Lesion number	White light diagnosis	Fluorescenceimaging	Tumor MFI/Background MFI	TBR
1	M/55	pT3bN0	Moderately differentiated squamous cell carcinoma		1	+	+	56.78/17.21	3.30
2	M/51	pT3aN2	Infiltrating high-grade urothelial carcinoma	HGI	2	+	+	45.33/19.09	2.37
3	M/82	pT1N0	Noninvasive high-grade papillary urothelial carcinoma	HGN	3	+	+	51.23/14.72	3.48
4	M/67	pT2aN0	Infiltrating high-grade urothelial carcinoma with squamous differentiation	HGI	4	+	+	48.29/15.93	3.03
5	M/63	pT2bN0	Infiltrating high-grade urothelial carcinoma with squamous differentiation	HGI	5	+	+	49.48/22.19	2.23
6	M/76	pT4aN1	Invasive urothelial carcinoma involving prostate	HGI	6	+	+	79.30/26.41	3.00
7	M/75	pT2aN0	Infiltrating high-grade urothelial carcinoma	HGI	7	+	ICG	42.45/36.28	1.17
8	F/72	pT2bN0	Infiltrating high-grade urothelial carcinoma	HGI	8	+	+	53.29/13.85	3.84
9	M/68	pT1N0	Noninvasive papillary urothelial carcinoma	HGN	8	+	+	58.35/17.39	3.35
10	F/66	pT3bN1	Invasive urothelial carcinoma	HGI	10	+	ICG	41.28/39.73	1.04
11	M/75	pT3bN0	Infiltrating high-grade urothelial carcinoma	HGI	11	+	+	47.29/13.98	3.38
12	M/66	Pt2aN0	Invasive urothelial carcinoma	HGI	12	+	+	57.39/17.97	3.19
13	M/77	pT1N0	Infiltrating high-grade urothelial carcinoma	HGN	13	+	+	54.38/15.73	3.45
14	M/59	pT2bN0	Infiltrating high-grade urothelial carcinoma	HGI	14	+	+	49.28/13.84	3.56
			CIS	CIS	15	+	+	47.76/14.92	3.20
15	M/69	PT1N0	Noninvasive high-grade urothelial carcinoma	HGN	16	+	+	61.29/21.73	2.82
16	F/72	pT3bN2	Invasive urothelial carcinoma	HGI	17	+	ICG	53.39/48.47	1.10
17	M/73	pT2aN0	Invasive urothelial carcinoma	HGI	18	+	+	59.48/21.29	2.79
			CIS	CIS	19	–	+	57.83/18.24	3.17
18	M/65	Pt3bN0	Infiltrating high-grade urothelial carcinoma with squamous differentiation	HGI	20	+	+	55.71/14.38	3.87
19	M/43	pT2aN0	Highly differentiated squamous cell carcinoma		21	+	+	61.33/21.49	2.85
20	M/72	pT3bN0	Invasive urothelial carcinoma, some of which are sarcomatoid carcinoma	HGI	22	+	+	51.39/18.36	2.80
			CIS	CIS	23	–	+	49.41/16.96	2.91
21	M/64	pT3bN0	Infiltrating urothelial carcinoma	HGI	24	+	+	74.22/24.45	3.03
22	M/65	PT2bN0	Infiltrating urothelial carcinoma	HGI	25	+	+	65.32/18.81	3.47
23	M/63	pT2aN0	Infiltrating urothelial carcinoma	HGI	26	+	+	58.39/18.72	3.12
24	M/58	PT1N0	Infiltrating urothelial carcinoma	HGI	27	+	+	49.73/16.76	2.97
			CIS	CIS	28	+	+	47.72/16.54	3.16
25	M/60	PT2bN0	Infiltrating urothelial carcinoma	HGI	29	+	+	52.18/15.39	3.39

## 4 Discussion

Blockade of CD47 function (a universally expressed molecule in all cancers) results in tumor cell phagocytosis and elimination (17). Research has shown that CD47 is expressed in all human bladder tumors, most notably in CD44+ cells (18). Furthermore, CD47 is highly expressed in NMIBC and MIBC compared with the terminally differentiated luminal umbrella cells of normal human bladder urothelium (19). Our previous research has shown that anti-CD47 is more effective in the identification of BC cell lines compared with other molecular tracers (e.g., NYZL1, PLZ4, and CA9 antibodies). CD47-targeted optical molecular imaging has demonstrated high diagnostic accuracy for BC detection and usefulness in the resection of such tumors (20). Another study has shown that the contrast of images was obviously intensified under CD47-targeted NIR molecular imaging, favoring the real-time diagnosis of upper urinary tract urothelial carcinoma (21).

ICG is a NIR fluorescent dye approved by the U.S. Food and Drug Administration. It does not present any detail regarding targeting tumor tissue. ICG is utilized in clinical practice for the visualization of the vascular system or lymphatic vessels (22–24). While ICG has a small amount of fluorescence in an aqueous solution, its emission intensifies with the combination of protein hydrophobic pockets (e.g., albumin) or cell membrane. We employed an ICG-anti-CD47 construct to target BC in human specimens. *Ex vivo* molecular imaging of 25 BC samples (including two patients with bladder squamous cell carcinoma) illustrated that fluorescent tracer dependent on anti-CD47 targeting guidance attached to CD47 molecules on the surface of tumor cells. Under NIR light, the fluorescence intensities of BC and normal urothelium were notably distinct (MFI: 55.47 ± 8.16 vs. 17.94 ± 3.32, respectively; P < 0.001), supporting the real-time diagnosis of tumors. We found that optical molecular imaging technology significantly enhanced the contrast of visual images. This approach can improve the visualization of tumors and assist physicians in BC detection and treatment. Generally, using NIR optical molecular imaging technology, the qualitative evaluation of fluorescence signals in all patients demonstrated that the MFI in tumor tissues was higher than that recorded in normal background tissues.

The detection of flat, small malignant lesions, particularly CIS, on the bladder wall is difficult. CIS is a high-risk NMIBC constrained to the mucosa. Under WLC, this type of tumor presents a structure and appearance similar to inflammatory lesions, potentially leading to misdiagnosis. The latest European Association of Urology guidelines on NMIBC recommend the use of photodynamic diagnosis or narrow-band imaging as an auxiliary imaging mode for WLC during tumor biopsy and resection (25). This approach can improve the detection rate of papillary lesions and CIS. However, owing to its low specificity for tumors, it may lead to false positive results in BC detection, especially in the presence of inflammatory lesions, scar hyperplasia, and acute bleeding in the bladder (11, 26, 27).

An increased metabolism results in higher acidity of the tumor microenvironment (28). Under the condition of low extracellular pH, insertion of the pH low insertion peptide (pHLIP) into the cell membrane can accurately target acidic cells. In a previous study, 22 fresh intact bladder specimens have been intravesically infused with ICG pHLIP for the diagnosis of BC through targeted imaging. The sensitivity and specificity rates of this method were 97% and 100%, respectively (29). In another study using a mixture of polysaccharide cyanamide cyanine and pHLIP (ICG pHLIP), NIR molecular imaging has been performed on segregated upper urinary tract specimens obtained from 12 patients. Compared with an ordinary white light examination, more tumor lesions were detected by pHLIP-mediated NIR molecular imaging (detection rate: 78.9% vs. 100%, respectively) (30). Therefore, considering the heterogeneity of BC, the use of molecular markers that can classify various tumor features could facilitate the development of more accurate and personalized detection protocols. In the present study, through ICG-anti-CD47 NIR fluorescence imaging, we found and pathologically confirmed two cases of bladder CIS that were not previously detected by cystoscopy.

This study comprised a mixture of different subtypes of urothelial carcinoma and squamous cell carcinoma, as expected given that the disease had advanced to the point where the bladder had to be removed. The cases included typical high-grade urothelial carcinoma but also had different variants with squamous cell differentiation or sarcomatoid features. It appears that the sensitivity (93%) and specificity (100%) of tumor targeting by ICG-anti-CD47 is irrelevant to the subtype of tumor.

There are many studies on molecular markers of bladder cancer, Research shown that the expression of PD-1, PD-L1 and cAMP is able to predict mortality at 3 years in patients with locally advanced (pT3-4) and/or positive lymph nodes disease (31). In addition, Some studies have confirmed that the safety and efficacy of ICG technology and ICG guided NIRF is a very useful tool in pediatric minimally invasive surgery to perform a true imaged-guided surgery (32, 33). Optical molecular imaging has the potential to improve the detection rate of tumor lesions in bladder tissue samples. However, the available evidence is limited to preclinical studies, and several scientific and technical issues still need to be addressed during the clinical transition. For example, fluorescence signal intensity and TBR are important metrics to assess the clinical application value of optical molecular probes. however, even if a higher TBR can be obtained during the operation, the data acquisition may be affected by the region of interest selected by the operator. So, it difficult to conduct multicenter studies and compare experimental results from different research centers.

This study had several limitations. First, this *ex vivo* feasibility study included a small number of patients. Hence, the present findings should be verified in a study involving a larger sample size. Second, future studies should evaluate this approach in more pathological types of BC (e.g., adenocarcinoma, carcinosarcoma, etc.). However, given the low incidence rate of these pathological types, a longer period of specimen collection may be required. Finally, considering clinical transformation, the use of this target tracer in humans is necessary. Notably, anti-CD47 b6h12 is a murine monoclonal antibody that is not safe for use in humans. Nevertheless, a humanized monoclonal anti-CD47 (HU5F9-G4) is currently being investigated in phase I clinical trials for the treatment of advanced solid tumors and lymphomas (34).

By incubating freshly isolated bladder samples with ICG-anti-CD47 molecular fluorescent tracers, we tested the practicability of this new molecular target. The contrast of images obtained through CD47-targeted NIR molecular imaging was notably enhanced, contributing to the real-time diagnosis of BC. Thus, NIR molecular imaging based on CD47 is a promising diagnostic approach. However, multidisciplinary cooperation among urologists, chemists, physicists, and pharmacologists is necessary to achieve optimal clinical application of this method.

## 5 Conclusions

In conclusion, ICG-anti-CD47 has the potential to become a promising tool for the early detection of BC. It exhibits high sensitivity and specificity regardless of the tumor subtype. This method can be used to monitor the stage of disease and mark lesions for surgical resection. Nevertheless, clinical trials involving toxicological and pharmacological studies are warranted to investigate the safety profile of the ICG-anti-CD47 imaging agent. Based on the present findings, this agent may optimize BC diagnosis and resection. Consequently, the recurrence rate may be reduced, the prognosis of patients may be improved, and the medical costs for BC treatment may decline. Additionally, the achievement of targeted imaging may guide the delivery of therapeutic molecules to bladder tumor cells, providing opportunities for targeted therapy of BC.

## Data availability statement

The raw data supporting the conclusions of this article will be made available by the authors, without undue reservation.

## Ethics statement

The studies involving human participants were reviewed and approved by the Ethics Committee of First Hospital of Shanxi Medical University. The patients/participants provided their written informed consent to participate in this study.

## Author contributions

(I) Conception and design: HH and XY. (II) Administrative support: GC and XY. (III) Provision of study materials or patients: HH, XW, GC and XY. (IV) Collection and assembly of data: HH and YQ. (V) Data analysis and interpretation: HH. (VI) Manuscript writing: All authors. (VII) All authors contributed to the article and approved the submitted version.
